# Refugee stigma and its toll on mental health: development and validation of the refugee stigma scale (RSS)

**DOI:** 10.1136/bmjgh-2024-017276

**Published:** 2025-11-13

**Authors:** Hamed Abdollahpour Ranjbar, Khaled Elazab, Ibrahim Yigit, Fatema Almeamari, Ceren Acartürk, Gülşah Kurt, Janet Turan, Andrea Norcini Pala, Bulent Turan

**Affiliations:** 1 Department of Psychology, College of Social Sciences and Humanities, Koç University, Istanbul, Türkiye; 2 Department of Psychology, Faculty of Humanities and Social Sciences, Istinye Universitesi, Istanbul, Türkiye; 3 Department of Psychology, Clark University, Worcester, Massachusetts, USA; 4 College of Nursing, Florida State University, Tallahassee, Florida, USA; 5 School of Clinical Medicine, University of New South Wales, Sydney, NSW, Australia; 6 School of Public Health, The University of Alabama at Birmingham, Birmingham, Alabama, USA; 7 School of Public Health, SUNY Downstate Health Sciences University, New York City, New York, USA

**Keywords:** Mental Health & Psychiatry, Global Health, Public Health, Health policies and all other topics

## Abstract

**Background:**

Unprecedented, forced displacement, especially from conflict and war areas, requires addressing resultant mental health issues.

**Aim:**

Refugees experience mental and physical health problems due to post-displacement stressors, and the pervasive stigma associated with refugee status can exacerbate these difficulties, highlighting the need for a comprehensive assessment tool to understand various facets of refugee stigma.

**Method:**

We developed the refugee stigma scale (RSS), consisting of 43 items informed by the literature, qualitative and quantitative data. The scale includes four theoretical dimensions of stigma: perceived community stigma, experienced stigma, anticipated stigma and internalised stigma. To examine convergent validity, validated self-report measures assessing depression, anxiety, post-traumatic stress disorder (PTSD), somatic symptoms (SSs), post-migration difficulties and contact experiences were used. Confirmatory factor analysis (CFA) examined the scale structure, and multiple-group CFA (MG-CFA) was used to assess measurement invariance. Cronbach’s alpha was used to test internal reliability, and associations of the stigma dimensions with depression, anxiety, PTSD and SSs were examined to test validity.

**Results:**

In a sample of (n=851, 404 Syrian, 63.9% men; 447 Afghan, 67.1% men) refugees in Türkiye, the CFA supported the hypothesised four-factor structure of the RSS (fit indices: χ^2^=4051.880, df=1169, p<0.001, comparative fit index=0.99, Tucker-Lewis index=0.99, root mean square error of approximation=0.054). MG-CFA suggested that RSS is invariant across Syrian and Afghan refugees, gender, educational level, length of stay and legal status in the host country. High internal reliability (α>0.88) and strong associations of the stigma dimensions with health outcomes support the reliability and convergent validity of the RSS.

**Conclusion:**

This study provides robust evidence for the RSS as a scale assessing different dimensions of stigma related to refugee status. The RSS can provide valuable insight into the complex web of refugee status stigma and mental and physical health difficulties.

**WHAT IS ALREADY KNOWN ON THIS TOPIC**

- Refugees frequently experience severe stigma, which has a negative impact on both their mental and physical health. While stigma has been researched in a variety of settings, there hasn’t been a comprehensive instrument to gauge the particular stigma faced by refugees.

**WHAT THIS STUDY ADDS**

- This research introduces and validates the refugee stigma scale (RSS), the first all-inclusive instrument developed to gauge various aspects of stigma particularly associated with being a refugee. The scale illustrates how stigma affects Syrian and Afghan refugee populations’ psychological and physical well-being.

**HOW THIS STUDY MIGHT AFFECT RESEARCH, PRACTICE OR POLICY**

- With the use of the RSS, researchers could more accurately examine the multidimensional stigma that refugees face and its effects on both physical and mental health. By directing focused treatments that specifically address stigma-related issues, practitioners can improve the well-being of refugees by using the RSS. The knowledge acquired from this tool can help shape policies that will, in turn, improve the way that refugee populations are supported at the policy level by assisting in the fight against stigma, encouraging social inclusion and minimising health disparities.

## Introduction

 Over 110 million people have been forcibly displaced worldwide, as recorded in June 2023.^1^ Of these displaced people, a staggering number—more than 35 million—are refugees.^2^ Syrian and Afghan refugees are among the top refugee populations. The majority of refugees who grapple with mental health problems are not able to obtain the proper mental health services.^3^ Many refugees have been exposed to a wide range of potentially traumatic experiences and human rights violations such as torture, witnessing the death/injury of a loved one, separation and arrests. In addition, they experience a range of post-displacement stressors once they reach a host country, such as impoverishment, limited access to basic services, unemployment and social isolation.^4 5^ These cumulative stressors have detrimental effects, putting conflict-affected refugee populations at a heightened risk of developing mental health problems, including post-traumatic stress disorder (PTSD), depression and anxiety.^4 6 7^

Moreover, mental health problems are more prevalent among conflict-affected individuals than in the general population.^8^ A meta-analysis found prevalence rates of 31.46% for PTSD, 31.5% for depression and 11% for anxiety disorder among refugees and asylum seekers.^9^ Similarly, a recent study among Syrian and Afghan refugees and asylum seekers in Türkiye (formerly known as Turkey) reported a high prevalence of mental health problems, ranging from 41.1% to 50.3% for depression, 39.6% to 41% for anxiety and 41.6% to 46.5% for PTSD symptoms, respectively.^10^ Beyond traumatic experiences prior to, during or after migration and associated poor mental health, post-migration stressors that involve prejudice, stigma and discrimination in host countries can exacerbate negative mental and physical health outcomes (e.g., ^11^). Crucially, recent research, exemplified by the ecological model of refugee distress proposed by Miller and Rasmussen,^12^ suggests that the mental well-being of refugees and asylum seekers is influenced not solely by past experiences of war but also by a multitude of persistent stressors within their social environment.^12^ In other words, mental health challenges in this population are intricately connected to the ongoing stressors associated with displacement. Therefore, while acknowledging the role of past trauma, examining the impact of current stigma and discrimination experiences related to living as a refugee might offer a new avenue to understand refugee mental and physical health, which is essential for the well-being and future of both individuals and society.

### Stigma and health

Stigma arises when an attribute attached to a specific group of individuals renders them inferior and socially undesirable in the eyes of society.^13^ This attribute can be linked to a person’s racial/ethnic background, illness, or any other characteristic subject to labelling and categorising based on social perceptions. Stigmatised individuals are perceived as having a lower social value than others, which contributes to increased experiences of prejudice, discrimination and ostracism.^13^,^15^

Over the years, stigma has been conceptualised using various theoretical perspectives, which has provided valuable insights into the different aspects and social impacts of stigma. Jones proposed six dimensions of stigma, namely concealability (the degree to which a stigmatised condition is observable), course (if the condition is perceived as provisional or enduring), disruptiveness (the extent the condition interrupts social interactions), aesthetics (how unappealing the condition is perceived to be), origin (perceived cause or responsibility for the condition) and peril (the perception of danger associated with the condition).^16^ Similarly, Elliot^17^ theorised stigma as a mechanism of social control that maintains systems of exclusion and power. Later, Link *et al.*^18^ proposed their modified labelling theory, which proposes that expectations in the socio-cultural environment around stigma can cause self-stigmatisation, which is ultimately a contributing factor in worse mental health outcomes. These frameworks bring together the threads of stigma, societal attitudes and personal agency, elucidating the layers of experience and contributing to ongoing dialogues in this critical area of research.

Stigma from the perspective of stigmatised individuals is conceptualised in terms of different dimensions, including internalised stigma (i.e., acceptance of negative characterisations in society and applying these to oneself), perceived community stigma (i.e., perceptions of the existence and severity of stigmatising attitudes and discrimination in the community against individuals with the stigmatised condition or identity), experienced stigma (i.e., facing acts of discrimination and prejudice), and anticipated stigma (i.e., expectation of being treated negatively).^19 20^ A substantial body of research suggests that different dimensions of stigma affect the emotions, cognitions and behaviour of stigmatised individuals (e.g., people living with HIV, sexual and gender minorities, patients with cancer, substance users and people with schizophrenia). This may result in distinct adverse psychosocial and physical health outcomes, including higher depression, increased anxiety and lower social support.^21^ Additionally, stigma was found to contribute to lower self-efficacy^22^ and non-adherence to medical treatment.^20 23^

To measure stigma, several scales have been developed to assess various dimensions of stigma experiences among different populations. One of these tools is the internalised stigma of mental illness (ISMI) Scale,^24^ which assesses how people with mental illness internalise societal stigma and how it affects their well-being. The stigma and self-stigma scales^25^ assess both external stigma (i.e., societal attitudes) and self-stigma, which involves the internalisation of these beliefs. Other scales have also been developed specifically to address stigma in particular populations. For instance, the HIV Stigma Scale^14^ assesses experiences of stigma by people living with HIV regarding personalised stigma, disclosure concerns, negative self-image and public attitude concerns. Another similar scale is the self-stigma of mental illness scale,^26^ which explores internalisation, measuring the degree to which individuals with mental illness are aware of stereotypes about them, whether they agree with the stereotypes and believe the stereotypes apply to them, and whether they feel that their self-esteem or self-worth is diminished by the stereotypes.

### The stigma of being a refugee

The entire relevance of stigma as a basic driver of population health has been disguised by the fact that bodies of research relevant to certain stigmatised statuses have generally emerged in different domains and have concentrated on single outcomes at one level of analysis.^27^ In the case of stigma against refugees, social and political factors are interlinked with the attitudes of host communities. These attitudes consequently shape refugees’ experiences after displacement and the interactions between the refugees and hosting community members.^28^ Negative stereotypes about outgroups act as significant drivers of various emotions (eg, anxiety, disgust, pity, anger) and behavioural tendencies (prejudice, social distance, support of restrictive policies).^29^ Earlier research highlighted such negative attitudes toward refugees in various countries, including Türkiye (e.g., ^30 31^). For instance, a recent population-based survey suggested Turkish participants have negative perceptions and worries about Syrian refugees.^32^ Findings suggest that many in Turkish society associate labels such as ‘dangerous’, ‘rude’, ‘lazy’ or ‘beggars’ with Syrian refugees and report reluctance to be in close contact with them, indicating a desire for refugees to return to their home country. In addition, recent qualitative research highlights the prevalence of negative sentiments, marginalisation and instances of racial discrimination against Afghan migrants in Türkiye.^33^ Likewise, refugees describe the stigma of being a refugee (being labelled as ‘untrustworthy’, ‘stupid’ and ‘dangerous’) and its negative impacts on their sense of belonging.^34 35^

Refugees are often viewed as socially inferior (for review see;^28^), which may lead to continued stress, exclusion and poorer health outcomes.^36^ While stigmatisation has been studied among forced migrants, extant studies have mostly been qualitative investigations,^37 38^ or on perceived discrimination among forced migrants (mostly due to ethnicity or having mental health problems, not stigma due to being a refugee specifically).^39^ Discrimination is only one of several dimensions of stigma experienced by refugees.^40 41^ Thus, it is critical to identify and address the many difficulties resulting from the stigmatisation that refugees experience. Currently, there is a lack of comprehensive measurement tools to assess different dimensions of stigma related to living as a refugee. In order to fill this gap, the refugee stigma scale (RSS) was meticulously developed in this study to thoroughly examine different dimensions of stigma about living as a refugee.

### The current study

Drawing on previous literature addressing stigma related to other attributes, conditions or identities (e.g., people living with HIV), we aimed to develop and test a multifaceted scale that measures various dimensions of stigma related to living as a refugee (i.e., internalised, perceived community, enacted or experienced and anticipated stigma) faced by Syrian and Afghan refugees. Subsequently, we examined the associations that these stigma dimensions have with a range of mental health-related outcomes, subjective physical health and other related experiences to establish the scale’s criterion validity. To the best of our knowledge, this study represents the first attempt to develop and establish a measure assessing all dimensions of stigma related to living as a refugee and to examine its potential impact on refugees’ health and well-being.

## Methods

### Refugee stigma scale (RSS)

The RSS was developed by our research group to assess dimensions of stigma related to living as a refugee. It consisted of 43 items rated on a 5-point Likert-type scale ranging from 1 (*Strongly disagree*) to 5 (*Strongly agree*), with higher scores indicating greater experiences of stigma due to being a refugee. Sample items included ‘*I feel ashamed of being a refugee/immigrant*’ (In our scale, we deliberately juxtaposed the terms ‘refugee’ and ‘immigrant’ as some participants did not hold official refugee status. Furthermore, we suggest that this scale possesses adaptability for utilisation with individuals characterised as ‘asylum-seekers’, contingent on the legal status of the population under investigation (internalised stigma), ‘*Most people seem uncomfortable with refugees/immigrants*’ (perceived community stigma), ‘*People stayed away from me because I am a refugee/immigrant*’ (experienced stigma) and ‘*People will stay away from me because I am a refugee/immigrant*’ (anticipated stigma). The items for each subscale are presented in table 1.

**Table 1:** Items and factor loadings for the four-factor model and internal reliability coefficients (ɑ) of the refugee stigma scale

Factor (subscale)	Item	b	SE	α
RSS				
Internalised stigma	(1) I feel ashamed of being a refugee/immigrant	0.788*	0.03	0.9
	(2) Being a refugee/immigrant makes me feel that I am a bad person	0.843*	0.032	
	(3) I feel I’m not as good as others because I am a refugee/immigrant	0.850*	0.03	
	(4) I think less of myself because I am a refugee/immigrant	0.872*	0.032	
	(5) I feel like it is my fault that I became a refugee/immigrant	0.563*	0.042	
	(6) I deserve bad things for being a refugee/immigrant	0.634*	0.043	
	(7) I feel less worthy because I am a refugee/immigrant	0.869*	0.033	
	(8) I feel inferior to people who are not refugees/immigrants	0.813*	0.032	
Perceived stigma	(1) Most people seem uncomfortable with refugees/immigrants	0.741*	0.026	0.95
	(2) Most people view refugees/immigrants as immoral	0.811*	0.027	
	(3) Most people believe that refugees/immigrants should be isolated from society	0.804*	0.03	
	(4) Most people don’t want to be friends with a refugee/immigrant	0.847*	0.031	
	(5) Most people don’t want their children around refugees/immigrants	0.846*	0.031	
	(6) Most people believe that refugees/immigrants are a threat to society (eg, taking away jobs, taking advantage of resources, changing culture)	0.814*	0.028	
	(7) Most people believe refugees/immigrants are dangerous/criminal	0.866*	0.031	
	(8) Most people think that refugees/immigrants are inferior	0.883*	0.031	
	(9) Most people think that refugees/immigrants cannot be trusted	0.888*	0.032	
	(10) Most people think that refugees/immigrants should be blamed for their conditions	0.839*	0.031	
	(11) Most people feel that being a refugee/immigrant is a sign of personal failure	0.818*	0.034	
	(12) Most people believe that refugees/immigrants are lazy	0.753*	0.034	
Experienced stigma	(1) People stayed away from me because I am a refugee/immigrant	0.770*	0.03	0.93
	(2) I was denied access to certain areas because I am a refugee/immigrant	0.724*	0.031	
	(3) People made fun of me due to being a refugee/immigrant	0.818*	0.027	
	(4) I was blamed for being a refugee/immigrant	0.826*	0.026	
	(5) I was denied opportunities in the workplace/school (eg, acceptance, extracurricular activities, promotion, raise, opportunities to work with customers) because I am a refugee/immigrant	0.740*	0.031	
	(6) I was treated badly by doctors and/or nurses in healthcare settings because I am a refugee/immigrant	0.716*	0.032	
	(7) I was made to feel unwelcome by Turkish people because I am a refugee/immigrant	0.854*	0.028	
	(8) People acted as if I were not smart because I am a refugee/immigrant	0.822*	0.028	
	(9) People acted as if they were afraid of me because I am a refugee/immigrant	0.821*	0.027	
	(10) People acted as if I were dishonest because I am a refugee/immigrant	0.838*	0.028	
	(11) I was insulted because I am a refugee/immigrant	0.828*	0.028	
	(12) I was stared at or pointed at (looked at intently) in public because I am a refugee/immigrant	0.723*	0.03	
Anticipated stigma	(1) People will stay away from me because I am a refugee/immigrant	0.804*	0.024	0.94
	(2) People will make fun of me due to being a refugee/immigrant	0.827*	0.023	
	(3) People will think it is my fault that I became a refugee/immigrant	0.738*	0.027	
	(4) I will be denied opportunities in the workplace/school (eg, acceptance, extracurricular activities, promotion, raise, opportunities to work with customers) because I am a refugee/immigrant	0.738*	0.026	
	(5) I will be treated badly by doctors and/or nurses in healthcare settings because I am a refugee/immigrant	0.762*	0.027	
	(6) I will be made to feel unwelcome by Turkish people because I am a refugee/immigrant	0.839****	0.024	
	(7) People will act as if I am not smart because I am a refugee/immigrant	0.843*	0.024	
	(8) People will act as if they are afraid of me because I am a refugee/immigrant	0.814*	0.023	
	(9) People will act as if I am dishonest because I am a refugee/immigrant	0.855*	0.023	
	(10) I will be insulted because I am a refugee/immigrant	0.850*	0.023	
	(11) I will be stared at or pointed at (looked at intently) in public because I am a refugee/immigrant	0.750*	0.026	

We have incorporated both terms ‘refugee’ and ‘immigrant’ within our items to encompass and address the experiences of both displaced populations.

* p<0.001.

b, standardised factor loadings; RSS, Refugee Stigma Scale; α, Cronbach’s alpha for each subscale.

### RSS development process

We created an initial pool of 61 items informed by: (1) Qualitative data (in-depth interviews and focus groups) from refugees in Istanbul that we used to identify stigma dimensions and item wording for stigma related to being a refugee, (2) Previous research and literature on stigma related to being a refugee or a migrant living in a foreign country, (3) Data from a study we recently conducted (Kurt et al.),^42^ where we collected open-ended statements from Turkish people regarding perceptions of Syrian people living as refugees in Türkiye and (4) Items in existing measures assessing other types of stigma, such as stigma based on race,^43^ and migration stress,^44^ HIV,^14^ mental health^45 46^ and perceived discrimination.^47^ Using these sources, we developed subscales measuring (a) Experienced stigma, (b) Perceived community stigma, (c) Internalised stigma and (d) Anticipated stigma. The research team then went through the items, selected the final pool of acceptable items for each stigma dimension and finalised measures of stigma related to living as a refugee, consisting of 50 items in total.

The items were then translated into Arabic and Dari by native translators with psychology degrees and research backgrounds (using the back-translation method: Arabic and Dari versions were back-translated into English by a different translator). The original English version was compared with the back-translation to finalise the wording.^48^ Next, we conducted cognitive interviews to evaluate the pool of created items from the perspective of potential participants.

#### Cognitive interviews

These interviews were held with 10 Syrian and 10 Afghan refugees to evaluate the initial pool of items. We recruited this group considering the same inclusion criteria used in the main study. The interviews were conducted in Arabic and Dari by experienced interviewers trained by the research team. Cognitive interviews are based on principles of cognitive psychology and are used to examine cognitive processes that respondents use when responding to questionnaire items.^49^

A detailed interview guide was prepared to ensure a degree of standardisation in each cognitive interview. To test the usefulness of the interview guide, cognitive interviews were initially piloted with two Syrian refugees and two Afghan refugees. Then, a total of 20 cognitive interviews were conducted, which, according to previous research,^49^ suffices to identify and test the items’ logic, clarity and acceptability. The recruitment process involved collaboration with local refugee organisations and community leaders who helped identify potential participants. Efforts were made to include a diverse group in terms of gender and age. The Afghan group consisted of seven women and three men, while the Syrian group consisted of an equal number (five) of men and women. The ages of participants ranged from 20–55. Each cognitive interview lasted approximately 40 min. The interviews were audio-recorded using digital recorders, and the audio files were uploaded to an encrypted, password-protected computer. The study team coded these data, selected the final pool of acceptable items and refined the wording of the items, thereby finalising the scales. Given the sensitivity of the subject matter, particular attention was paid to potential emotional distress. The interview guide included prompts designed to minimise distress while still capturing meaningful data. We particularly noted the items that participants reported as making them feel anxious or disturbed and rephrased or removed them after discussions among the research team members. Furthermore, interviewers were educated in clinical psychology and trained to recognise signs of distress and respond appropriately; there were no such examples of emotional distress during any of the interviews as none of the questions revolved around recollection of specific migration-related traumatic events. Participants were told at the beginning that the interviews could be paused or discontinued as needed, with readiness to offer support and referrals to mental health services when needed. No such cases were recorded.

### Sample size

To determine the sample size for this study, which aimed to assess the factorial structure of a newly developed measure with a pool of 61 items using confirmatory factor analysis (CFA), we followed guidelines established for weighted least squares mean and variance (WLSMV) estimation. For a theoretically expected four-factor structure, expected moderate communalities and factor loadings ≥0.60, recent simulation studies^50 51^ recommend a minimum of 10–15 participants per item or per parameter. Thus, a sample size between 700 and 1000 was considered appropriate to reach adequate power (0.80), stable parameter estimates and reliable model fit indices.^52^

### Participants

Participants included refugees from Syria and Afghanistan residing in different cities in Türkiye. Inclusion criteria were being born in Syria or Afghanistan, having fled to Türkiye due to conflict/war/difficult circumstances in Syria or Afghanistan, being at least 18 years of age, speaking Arabic (for Syrians) or Dari (for Afghans), having finished elementary education, and living in Türkiye for at least the last 6 months.

### Procedures

To reach potential participants, we collaborated with two associations operating in Türkiye, namely the Afghan refugees solidarity association and the Refugee and Asylum seekers assistance and solidarity association. The Qualtrics system was used to distribute the online survey through flyers on social media platforms among Syrians and Afghans via the aforementioned organisations. To maintain the integrity of voluntariness in participation, we limited the role of refugee agencies to only advertising our research flyers on their social media platforms, enabling participants to independently reach out to our research team without any influence or pressure from the agencies. Participants were compensated with grocery vouchers. The value of vouchers provided was set to be appropriate for the duration of participation: for interviewees who engaged in discussions lasting 40 min, we provided vouchers worth approximately *$6* USD, while individuals completing surveys, which took approximately 30–40 min, were offered the same amount. Our diverse research team, including local clinical psychologists and researchers with and without lived displacement experiences, co-designed the study through an iterative consultation process. This collaboration ensured cultural sensitivity, addressing sensitive issues and incorporating stakeholders’ perspectives in refugee mental health research in Türkiye.

### Ethical considerations

Participants provided online informed consent before participation. Participation was voluntary, and respondents could withdraw at any time. Data were collected anonymously, securely stored and used solely for research purposes. Participants were informed of their rights and any minimal potential risks. The study was approved by the Koç University Committee on Human Research Ethics (IRB code: 2021.037.IRB3.018).

### Patient and public involvement

Qualitative data from refugee interviews and focus groups in Istanbul informed our research question and outcome measures. Refugees also contributed to the study design through cognitive interviews that assessed item clarity, logic and acceptability, resulting in refinements in wording and removal of potentially distressing questions. No refugees participated in recruitment or study conduct. The findings will be disseminated in seminars and shared with stakeholders so that findings can functionally reach relevant communities and organisations. The participation burden was reduced through short surveys (30–40 min) and the offer of grocery vouchers as remuneration.

### Measures

The Hopkins Symptoms Checklist (HSCL-25). The Hopkins Symptom Checklist (58-item version) was developed as a self-report symptom inventory to assess five underlying symptom dimensions—somatisation, obsessive-compulsive, interpersonal sensitivity, anxiety and depression.^53^ Later, the developers used a shorter version (i.e., 25 items) for assessing depression and anxiety symptoms and demonstrated its usefulness in assessing these symptoms.^54 55^ The measure includes 25 items, each rated on a 4-point Likert scale ranging from 1 (*not at all*) to 4 (*extremely*), with higher scores indicating higher levels of depressive and anxiety symptoms. Mollica *et al.* adapted the HSCL-25 and found it a reliable measure in assessing depression and anxiety among trauma survivors.^56^ The HSCL-25 has been widely used in conflict-affected populations and found to have good psychometric properties, including Syrian and Afghan refugees and asylum seekers in Türkiye.^10^ In the current study, Cronbach’s alpha was 0.95 for Syrians and 0.96 for Afghans.

The somatic symptom scale (SSS-8^57^). SSS-8 was used to assess the presence and severity of common somatic symptoms (SSs) (e.g., back pain, headaches, trouble sleeping). It includes eight items, which are rated on a 5-point response option (0=*Not at all*, 4=*Very* much), with higher scores reflecting higher SSs. It has been the most commonly used self-report measure of the perceived burden of common SSs.^57 58^ In the current study, Cronbach’s alpha was 0.88 and 0.90 for Syrians and Afghans, respectively.

The Short Form of PTSD Checklist for DSM-5 (PCL-5-S;^59^) is a self-report checklist containing four items designed to assess the presence and severity of PTSD symptoms rated according to DSM-5 criteria in the past month. The short form includes four items that assess symptoms related to hyperarousal, intrusion, avoidance and negative cognition. Participants respond to each item on a 5-point scale from 0 (not at all) to 4 (extremely), with higher scores indicating greater severity of PTSD symptoms. A sample item is: ‘*Do you suddenly feel or act like the stressful experience is happening again as if you are reliving it?*’ Cronbach’s alpha for the Syrian and Afghan refugee population living in Türkiye was reported to be 0.80 and 0.83, respectively.^10^ In the current study, Cronbach’s alpha for the Syrian and Afghan refugees was 0.76 and 0.85, respectively.

Post-migration living difficulties checklist (PMLDC^60^). The PMLDC was used to assess post-displacement stressors experienced by Syrian and Afghan refugees. We used the 17-item version (e.g., ^61^), each item rated on a 5-point Likert scale ranging from 0 (*not a problem*) to 4 (*a very serious problem*). It has well-established sound psychometric properties in conflict-affected populations, including Syrian and Afghan refugees in Türkiye.^10 62^ In this study, Cronbach’s alpha was 0.88 for Syrians and 0.90 for Afghans.

### Contact experiences

Four items^63^ tapping into contact quantity and quality of Syrians and Afghans with Turkish people were used. The item for contact quantity, ‘*How frequently do you have contact with Turkish people?*’ is rated on a 5-point Likert scale ranging from 1 (*never*) to 5 (*very frequently*). The item for contact quality is ‘*When you meet with Turkish people, in general, do you find the contact to be cooperative*?’ ‘*When you meet with Turkish people, in general, do you find the contact to be positive?*’ ‘*When you meet with Turkish people, in general, do you find the contact to be pleasant?*’ rated on a 5-point Likert scale ranging from 1 (*not at all*) to 5 (*very much*).^63^ In the current investigation, Cronbach’s alpha for the 4-item scale was 0.80 for Syrians and 0.75 for Afghans.

### Data analysis strategy

#### First step: confirmatory factor analysis

We employed the *lavaan* package^64^ in R to perform a CFA of the theoretical factorial model of the RSS (Four-Factor Model: experienced stigma, perceived community stigma, internalised stigma, anticipated stigma). We used WLSMV estimation to account for the ordinal nature of the Likert-type items.^65^ The model fit was assessed with the following indices: χ^2^/degree of freedom CMIN/DF—values >3.0 suggest a good model fit,^66^ the root mean square error of approximation (RMSEA ≤0.05 suggests a good fit),^66^ the comparative fit index (CFI),^67^ and Tucker-Lewis index (TLI),^68^ where values >0.95 indicate excellent model fit.

#### Second step: revision of the questionnaire

Another assessment and inspection of the items was performed in the second phase. Seven items were eliminated from the questionnaire due to lower factor loadings, lower inter-item correlations, conceptual irrelevance or statistical and conceptual overlap with other items (The questionnaire and instructions for its use are provided in the online supplemental appendix).

#### Third step: test of measurement invariance

The analytic strategy for the structural and measurement invariance across groups (i.e., Afghan and Syrian) was conducted for the RSS.^69^ The multi-group CFA (MG-CFA^70^) was used to examine configural, metric and scalar measurement invariance (in this order). Configural invariance was used to confirm if RSS would show a four-factor model (after revision) across the two groups (i.e., Afghan and Syrian), metric invariance examined whether item loadings were similar across both groups and scalar invariance examined whether item intercepts were the same across groups. For MG-CFA, we relied mainly on Standardised Root Mean Squared Residual (SRMR) values, which are more sensitive to lack of invariance in factor loadings than in intercepts or residual variances;^70^ the other fit indices may be less trustworthy during the WLSMV estimation.^71^ ΔCFI, ΔRMSEA and ΔSRMR values and Δχ2 tests are offered for comparing configural, metric and scalar invariance models in MG-CFAs. We primarily evaluated CFI, RMSEA and SRMR results, which were unaffected by the present study’s large sample size, but we also referenced the overall fit of these models for comparative juxtaposition. Cut-offs advised by Rutkowski and Svetina^72^ were employed, with ΔCFI ≲0.020 and ΔRMSEA ≲0.030 to indicate a good fit of metric invariance models compared with configural invariance models, and ΔCFI ≲0.010, ΔRMSEA ≲0.01 and ΔSRMR ≲0.010 used to indicate a good fit of scalar invariance models compared with metric invariance model.^72^,^74^

#### Fourth step: reliability analysis

We calculated Cronbach’s alpha to evaluate the internal consistency of the four subscales of the RSS.

#### Fifth step: convergent validity analysis

Employing diverse constructs to provide evidence of associations and impacts is considered sound methodological practice and is important for convergent validity.^75^ We used the HSCL-25 to measure depressive and anxious symptomatology, PCL-5-S to measure PTSD symptoms, SSS-8 to measure SSs associated with psychological distress and a PMLDC to assess post-migration challenges refugees experience. Moreover, a scale was integrated to measure contact experiences (CEs) with host country citizens, which evaluated interactions and experiences with local citizens. This approach has helped us examine the convergent validity of the RSS through correlations with constructs theoretically related to stigma.

## Results

### Descriptive statistics

The analytic sample consisted of 851 refugees residing in various cities across Türkiye, including 404 individuals from Syria (63.9% men) and 447 individuals from Afghanistan (67.1% men). Table 2 presents the descriptive statistics and demographic characteristics of the participants, providing an overview of key variables in the study. These descriptive findings are followed by the evaluation of measurement properties and the results of the primary analyses addressing the study hypotheses.

**Table 2:** Sociodemographic characteristics

	Afghan		Syrian		Total	
	n	%	n	%	n	%
Gender						
Male	298	66.9	260	64	558	65.6
Female	146	32.8	145	35.7	291	34.2
Other	1	0.2	1	0.2	2	0.23
Total	445	52.3	406	47.7	851	–
Age M(SD)	30.6 (8.8)		33.2 (8.3)			
Education level						
Primary school/junior high school	196	48.3	108	24.3	304	35.7
High school	137	33.7	162	36.4	299	35.1
University degree	68	16.7	156	35.1	224	26.3
Master’s or PhD	5	1.2	19	4.3	24	2.8
Current legal/residence status						
Asylum applicant	161	36	25	6.2	186	22
Asylum applicant but rejected	36	8.1	1	0.2	37	4
Humanitarian protection	21	4.7	6	1.5	27	3
Conditional refugee	47	10.5	3	0.7	50	6
Under temporary protection	71	15.9	344	85.1	415	49
Residency permit	49	11	5	1.2	54	6
Turkish citizenship	2	0.4	13	3.2	15	2
No status/registration	46	10.3	6	1.5	52	6
Other	14	3.1	1	0.2	15	2
Family monthly income						
None	33	8.1	187	42.1	220	25.9
Below 8500 TL*	104	25.6	142	32	246	28.9
8500 TL	180	44.3	94	21.2	274	32.2
8501–17 000 TL	77	19	8	1.8	85	10
17 001–25 500 TL	10	2.5	1	0.2	11	1.3
25 501–34 000 TL	2	0.5	1	0.2	3	0.4
34 001 TL and higher	0	0	11	2.5	11	1.3
Marital status						
Single	59	14.6	174	39.1	233	27.4
Married/cohabitant	318	78.7	219	49.2	537	63.1
Divorced/separated	19	4.7	15	3.4	34	3.9
Widowed	7	1.7	13	2.9	20	2.3
Religion						
Christian	3	0.7	10	2.2	13	1.5
Muslim	396	97.5	423	95.1	819	96.2
Atheist/agnostic	1	0.2	7	1.6	8	0.9
Other	6	1.5	5	1.1	11	1.3
Children						
Yes	329	81	244	54.8	573	67.3
No	77	19	201	45.2	278	32.7
Length of stay in Turkey						
6 months–1 year	1	0.2	32	7.2	33	3.9
1–3 years	3	0.7	104	23.4	107	12.6
3–5 years	25	6.2	155	34.8	180	21.2
5–7 years	80	19.7	107	24	187	22
7–9 years	169	41.6	30	6.7	199	23.4
10 years or more	128	31.5	17	3.8	145	17

* Minimum Türkiye wage at the time of assessment.

TL, Turkish Lira.

### Confirmatory factor analysis

**Figure 1:**
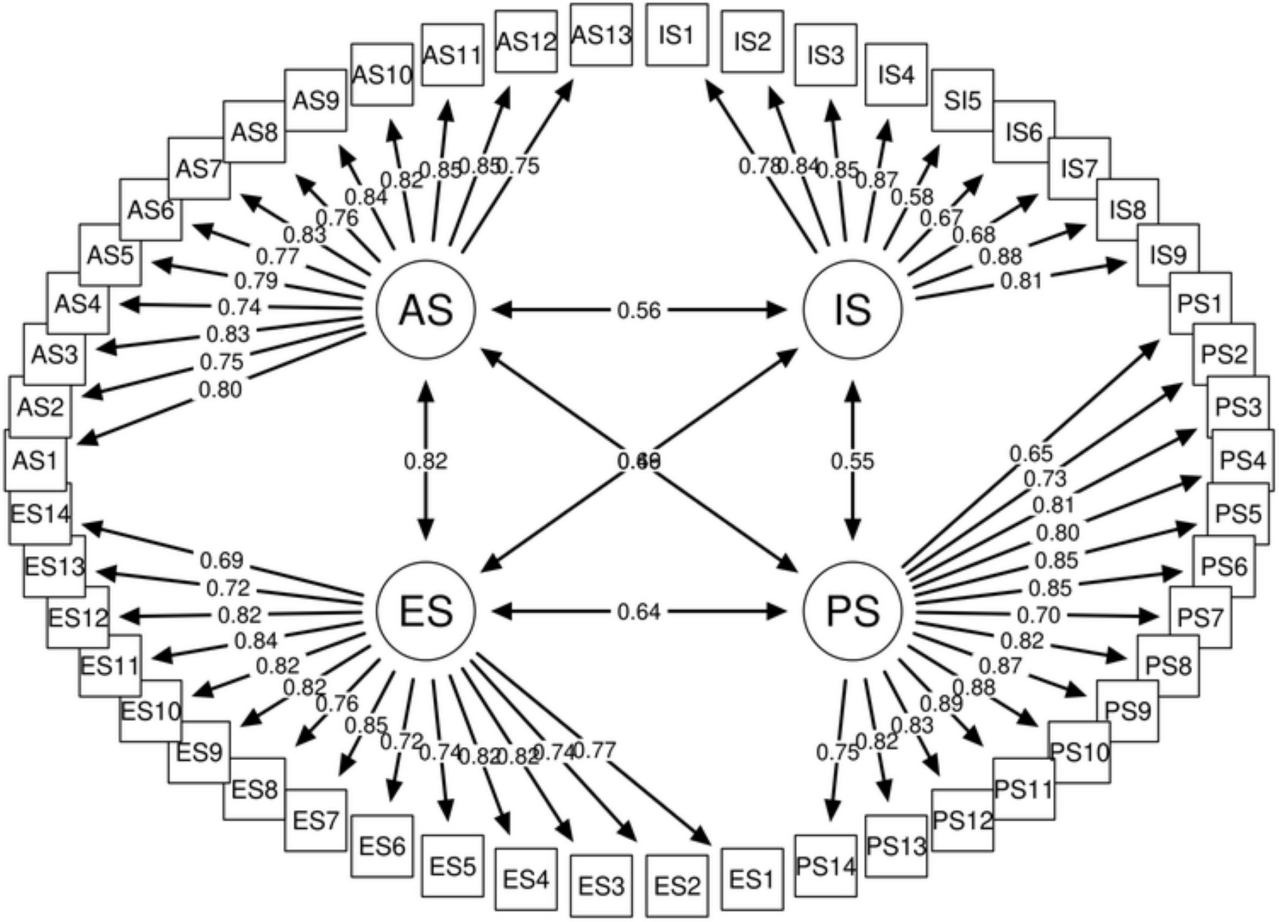
Factor loadings for the items of the refugee stigma scale. AS, anticipated stigma; ES, experienced stigma; IS, internalised stigma; PS, perceived community stigma.

Fit indices revealed that the CFA model fits the data well (figure 1). The χ^2^ statistic was (4051.880, df=1169, p<0.001). The χ^2^ statistic, however, is sample size sensitive and can be unduly sensitive in big samples.^52^ The CFI=0.99, TLI=0.99 and RMSEA=0.054 (95% CI (0.053 to 0.056)), on the other hand, suggested a satisfactory model fit.^76^

### Measurement invariance

We tested the cross-group measurement invariance for five major grouping variables, namely, culture (Afghans vs. Syrians), gender (men vs. women), education level (low vs. high), length of stay in Türkiye (short vs. long) and legal status (stable vs. unstable)—to examine whether the factor structure, factor loadings and intercepts of the RSS four-factor model were similar across groups (see table 3). Full measurement invariance (configural, metric and scalar) was established for culture and gender, suggesting that the RSS functions equivalently for Afghans and Syrians and for men and women, and thus valid comparisons of latent means between these groups are possible. Full measurement invariance was also supported for education level, length of stay and legal status. For education level, we classified participants into lower education (primary school to high school) and higher education (university degree or higher), which captures relevant differences in educational achievement. Similarly, the length of stay was categorised into short-term stay (i.e., < 5 years) and long-term stay (i.e., 5 years or more) to capture differences in adaptation and integration experiences. Ultimately, we classified humanitarian protection, Turkish citizenship and residency permits as stable legal statuses, while all other statuses were categorised as unstable. Importantly, these findings collectively provide strong evidence for the measurement invariance of the RSS across a range of demographic and contextual variables.

**Table 3:** Fit indices and χ^2^ difference tests for measurement invariance by culture, gender, education level and length of stay in Türkiye

Grouping variable	Model	χ² (df)	CFI	TLI	RMSEA (95% CI)	SRMR	ΔCFI	ΔRMSEA
Culture (Syrians vs Afghans)	Configural	3625.14 (1708)	0.994	0.994	0.052 (0.029 to 0.074)	0.049	—	—
	Metric	3986.78 (1747)	0.994	0.993	0.055 (0.032 to 0.079)	0.052	0.000	+0.003
	Scalar	4352.84 (1872)	0.993	0.993	0.056 (0.032 to 0.080)	0.049	−0.001	+0.001
Gender (men vs women)	Configural	4970.788 (2338)	0.995	0.994	0.051 (0.029 to 0.074)	0.050	—	—
	Metric	4401.098 (2384)	0.994	0.994	0.054 (0.031 to 0.078)	0.053	−0.001	+0.003
	Scalar	5116.559 (2530)	0.995	0.995	0.050 (0.028 to 0.072)	0.050	+0.001	−0.004
Education level (low vs high)	Configural	4865.337 (2338)	0.995	0.994	0.051 (0.028 to 0.073)	0.050	—	—
	Metric	4323.985 (2384)	0.994	0.993	0.054 (0.031 to 0.077)	0.052	−0.001	+0.003
	Scalar	5016.789 (2530)	0.994	0.995	0.049 (0.027 to 0.072)	0.050	0.000	−0.005
Length of stay in Türkiye (short vs long)	Configural	5044.423 (2338)	0.995	0.994	0.052 (0.029 to 0.074)	0.050	—	—
	Metric	5452.400 (2384)	0.994	0.993	0.056 (0.032 to 0.079)	0.053	−0.001	+0.004
	Scalar	5146.403 (2530)	0.995	0.995	0.050 (0.027 to 0.072)	0.050	+0.001	−0.006
Legal status (stable vs unstable)	Configural	4409.46 (2338)	0.994	0.994	0.053 (0.030 to 0.076)	0.050	—	—
	Metric	3986.21 (2384)	0.994	0.994	0.057 (0.033 to 0.080)	0.050	0.000	+0.004
	Scalar	4477.69 (2530)	0.994	0.995	0.050 (0.028 to 0.073)	0.050	0.000	–0.007

ΔCFI and ΔRMSEA represent changes in fit indices between models.

CFI, Comparative Fit Index; df, degrees of freedom; RMSEA, Root Mean Square Error of Approximation; SRMR, Standardised Root Mean Square Residual; TLI, Tucker-Lewis Index; χ², chi-square statistic.

### Reliabilities and concurrent validity of the RSS subscales

The items and Cronbach’s alpha values for each subscale are presented in table 1 and suggest good internal consistency for each subscale. Following CFA and reliability assessments, seven items were eliminated due to lower factor loadings and inter-correlations. Consequently, the final RSS comprised 43 items, distributed as follows: eight items for internalised stigma, 12 items for perceived community stigma, 12 items for experienced stigma and 11 items for anticipated stigma.

Stigma items adapted from multiple validated scales: internalised stigma: items #1–4 were adapted and contextualised from the *HIV Stigma Scale*.^14^ Perceived stigma: items #1 and #5 were taken from the *HIV Stigma Scale*, while items #6, #7 and #9 were adapted from the *Barcelona Immigration Stress Scale*^44^ and the *Public Perception of Mentally Ill People Questionnaire*.^46^ Experienced stigma: items #1, #3, #6 and #8 were drawn from the *Race-Related Stress Measure*,^77^ *HIV Stigma Scale* and *Perceived Discrimination Scale*.^47^ Anticipated stigma: items #1, #2, #5 and #7 were designed to parallel experienced stigma items and were adapted from the *Race-Related Stress Measure*, *HIV Stigma Scale* and *Perceived Discrimination Scale*.

Bivariate correlations between stigma subscales and theoretically relevant variables (i.e., depression, anxiety and SSs) are presented in table 4. All correlations were significant and of small to large effect size, supporting the validity of the scales. Online supplemental table 1 displays all the findings of the correlation analysis and shows the relationships between the stigma subscales (IS, PS, ES and AS), mental health outcomes (anxiety, depression), SSs, PMLD and CEs. The stigma subscales showed significant moderate to strong positive relationships with both anxiety and depression symptoms, suggesting that higher levels of stigma are linked to higher levels of psychological distress. In particular, anxiety showed association values ranging from 0.32–0.51, and depression ranging from 0.37–0.55 with the stigma subscales. Furthermore, a positive correlation was found between stigma and SSs (coefficients ranging from 0.25–0.45) as well as living difficulties after migration (coefficients ranging from 0.25–0.55). On the other hand, CEs had a negative correlation (coefficients ranging from −0.21–−0.47) with all stigma subscales, suggesting that more frequent and welcoming contact is linked to lower levels of stigma. These results suggest substantial negative effects of stigma on mental health and SSs.

**Table 4:** Bivariate correlations between stigma subscales and clinical measures

	1	2	3	4	5	6	7
IS	1						
PS	0.39**/0.52**	1					
ES	0.29**/0.48**	0.42**/0.67**	1				
AS	0.39**/0.55**	0.53**/0.70**	0.70**/0.77**	1			
Depression	0.37**/0.48**	0.27**/0.53**	0.46**/0.54**	0.37**/0.55**	1		
Anxiety	0.32**/0.43**	0.25**/0.45**	0.42**/0.49**	0.33**/0.51**	0.75**/0.76**	1	
SS	0.25**/0.30**	0.20**/0.42**	0.36**/0.39**	0.28**/0.45**	0.70**/0.74**	0.68**/0.70**	1
PTSD	0.27**/0.41**	0.28**/0.52**	0.44**/0.54**	0.35**/0.54**	0.74**/0.81**	0.62**/0.71**	0.57**/0.70**

The values before slash (/) belongs to Syrians, and the values after slash (/) belong to Afghans.

All study variables’ bivariate correlations (mean and SD) are provided in online supplemental table 1.

**Correlation is significant at the 0.01 level (2-tailed).

AS, Anticipated Stigma; ES, Experienced Stigma; IS, Internalised Stigma; PS, Perceived Community Stigma; PTSD, Post-Traumatic Stress Disorder, the values before; SS, Somatic Symptoms.

## Discussion

We developed the first measure to assess various dimensions of stigma related to living as a refugee. The new scale explores different dimensions, including experienced, perceived community, internalised and anticipated stigma. A meticulous review of pertinent items in stigma questionnaires across various fields culminated in the development of the RSS.

CFA was employed to validate the factor structure of the RSS. The scale demonstrated robust fit indices affirming its ability to capture theoretical constructs of experienced, perceived community, internalised and anticipated stigma among sampled refugee populations. Analyses also supported measurement invariance across the two cultural groups (Afghan and Syrian), gender, educational level, length of stay in the host country (Türkiye) and legal status (stable vs. unstable), which supports the cross-cultural utility of the RSS.^69^ Although there are numerous differences between the two cultural groups in terms of legal status (see table 2), other post-migration living conditions and perceptions by the host community, our findings underscore the applicability of the different stigma dimensions (i.e., internalising, perceiving, experiencing and anticipating) to both groups, enhancing the likelihood of the scale’s generalisability across different refugee populations. However, future research is needed to examine the structure and validity of the RSS in other refugee groups in other parts of the world.

High Cronbach’s alpha values on each RSS subscale suggest strong internal consistency. The scale’s convergent and criterion validity is supported by the correlations found between the RSS subscales and associated variables, including PMLDs, CEs, mental health indicators (depression, anxiety and PTSD), and SSs (see table 4 and online supplemental table 1). The associations between the subscales of the RSS and mental health indicators, such as anxiety, depression and PTSD symptoms, are essential to understanding the emotional consequences of the stresses that refugees encounter. Depression, anxiety and PTSD symptoms are prevalent mental health issues among refugees, which are frequently made worse by traumatic events and post-migration life challenges. Studies have indicated that extended exposure to these types of stressors, in the absence of appropriate coping strategies or support systems, can result in serious mental health problems.^78^ Understanding these mechanisms is essential to developing focused interventions to address mental health issues among refugees.^7^

It is crucial to address stigma as an important factor contributing to mental health inequalities among displaced populations, as suggested by the medium to strong correlations found between stigma dimensions and mental health outcomes in the current study. In this vein, Baranik *et al.*, in their mixed-methods investigation, found comparable results, linking the encounters of discrimination instances among refugees to increased levels of self-reported anxiety, depression and sleep disturbances.^37^ Quinn’s qualitative research on asylum seekers and refugees in Scotland suggested that the repercussions of migration on mental health, exacerbated by racial biases and challenges within the asylum process, are amplified by the presence of stigma and discrimination.^79^ Sadeghi’s^80^ research suggested that refugees are significantly impacted by discriminatory environments, reporting heightened feelings of threat, stigma, marginalisation and a sense of being continuously foreign. The pervasive stigma, shaped by social and cultural determinants such as fear, historical trauma, social isolation, racial prejudices and adverse cultural perceptions of mental health, markedly impacts these marginalised communities.

Furthermore, these effects on health and well-being stemming from stigma are often magnified by the interplay of various forms of discrimination. Intersectional stigma^81^ illuminates the coexistence of multiple stigmatised conditions or identities within an individual, leading to compounded vulnerabilities and more intricate impacts. English *et al.* underscore the substantial effects of intersectional stress among racial and sexual minorities, predicting elevated levels of depression and anxiety.^82^ These intersectional ramifications can extend to refugee populations, where stigma intersects with other facets of their lives. Discrimination often manifests in multiple forms, particularly affecting marginalised groups susceptible to multiple types of stigma based on their social identities. Intersectional stigma may be observable, for instance, among minority ethnic groups with mental illness, facing both racism and the stigma surrounding mental health.^83^ In many communities, racism and other forms of discrimination have increased the stigma attached to mental health problems, exacerbating feelings of marginalisation.^84^

### Internalised stigma

While there is a dearth of literature specifically exploring dimensions of refugee stigma and their impact on mental health outcomes, the established associations between these stigma dimensions in other contexts and mental health adversities form a solid foundation for our findings. For instance, regarding our findings concerning the connections between internalised stigma and mental health problems, existing literature consistently suggests a strong negative association between the ISMI and psychosocial adjustment (such as hope and self-esteem), as well as treatment adherence.^20 85 86^ Similarly, associations between internalised stigma of HIV and the presence of depression, anxiety and feelings of hopelessness have been reported.^87 88^ Additionally, research highlights associations between internalised sexual stigma among heterosexual individuals and mental health concerns,^89^ among other findings.

### Perceived community stigma

Our findings regarding perceived community stigma and its associations with mental health outcomes are also analogous with literature in other contexts. For instance, Nam *et al.* found that perceived social stigma (using a social stigma questionnaire) plays a crucial role in amplifying the impact of trauma exposure during the pre-resettlement phases among refugees.^90^ Additionally, Latalova *et al* found that the perception of stigmas associated with health impacts the inclination to seek assistance and fosters a less favourable outlook on mental health interventions, leading to heightened levels of depression.^91^ Similarly, multiple studies report higher rates of depressive symptoms among individuals with higher perceived health stigma.^91^,^93^ Other studies also highlight the importance and associations of stigma related to different conditions with mood and anxiety disorders,^94 95^ generalised anxiety disorder^96^ and functional somatic syndrome.^97^

### Anticipated and experienced stigma

Finally, our results concerning experienced and anticipated stigma and their links to mental health outcomes align with existing literature in various contexts. Studies suggest that experienced stigma is positively associated with depression and anxiety,^98^ adverse outcomes in schizophrenia^99^ and negative outcomes among children with obesity, such as social isolation and decreased physical activity.^100^ On the other hand, anticipated stigma is associated with lower treatment adherence self-efficacy, higher depressive symptoms, higher coping by substance use,^101^ lower quality of life among people living with chronic conditions^102^ and lower functioning among individuals with depression.^103^

### Limitations and future directions

Although this study offers a thorough investigation of the stigma dimensions of being a refugee, several limitations should be considered. The utilisation of self-report measures has an inherent risk of self-report bias, which might impact the precision of answers about experiences of stigma and mental health markers. It is also important to consider the possibility of selection bias, as only specific groups with perhaps unique characteristics (being Syrian and Afghan refugees) were participants. This means there may be limits to the generalisability of the findings to the general refugee population. Background features (culture, language, rituals, etc.) and current contextual factors such as the legal status, support systems and socioeconomic conditions can significantly vary between Syrian and Afghan refugees in Türkiye. Future research needs to focus on the extent of stigma faced by these groups and how and to what degree stigma impacts their mental and physical health outcomes. In addition, research should investigate these dynamics among diverse refugee populations with differing contexts, living in different locations around the world. Future research is needed to test the usefulness of the RSS in other cultural contexts and geographical locations. While the cognitive interviews provided valuable insights, there were also important limitations to consider. The relatively small sample size, although acceptable as per previous studies, may not fully cover the diversity of experiences within Syrian and Afghan refugee populations. Furthermore, the focus on self-reported experiences introduces the possibility of social desirability bias. Additionally, because these experiences are prone to chronological and contextual fluctuations, the snapshot structure of the study may not adequately reflect the dynamic features of stigma and psychological wellness/deterioration among refugees. The focus of the study on mental health conditions, with an emphasis on depression, anxiety, PTSD and SSs, can overlook other important conditions related to other high-burden conditions, such as alcohol and substance use disorders among refugees. Furthermore, the study cannot account for the bidirectionality of the stigma–mental health relationship. Future research should focus on the drivers of refugee substance use and other mental health outcomes while also accounting for the interplay and interconnectedness of these variables. Furthermore, due to the cross-sectional and observational nature of the study, no conclusion about causality can be made. This warrants further research examining these effects experimentally and/or longitudinally to understand causal effects that are predicted on theoretical grounds.

## Conclusions

The present investigation of the various facets of stigma related to living as a refugee provides new insight into the complex relationship between refugee stigma and mental health consequences. This study fills a gap in the literature on refugees by situating the study within a framework that includes internalised, perceived community, experienced and anticipated stigma. These results not only suggest how susceptible refugees are to the negative impacts of stigma, but they also stress how urgently tailored interventions and support networks informed by research are needed to address stigma and discrimination against refugees. Through recognition of stigma’s ubiquitous and complex impact on mental health, the study opens the door to more sophisticated and focused strategies to empower and assist refugee communities in their quest for mental health and social integration. The scientific knowledge, including subscales generated in this study, can be applied in future research on different forced migrant groups and contexts to understand and develop programmes and interventions to mitigate the negative effects of stigma related to refugee status for displaced individuals and communities.

## Supplementary material

10.1136/bmjgh-2024-017276 online supplemental file 1

10.1136/bmjgh-2024-017276 online supplemental table 1

## Data Availability

Data are available upon reasonable request.
