# Supplementary material for: Refugee stigma and its toll on mental health: development and validation of the refugee stigma scale (RSS)
Source: BMJ Glob Health. 2025 Nov 13;10(11):e017276. doi: 10.1136/bmjgh-2024-017276 (PMC12625852; doi:10.1136/bmjgh-2024-017276)
Supplement: online supplemental file 1 [file bmjgh-10-11-s001.pdf]

Appendix.

<table>
<tr><th></th><th>Subscale</th><th>Item</th></tr>
<tr><td rowspan="14">Refugee Stigma Scale</td><td rowspan="8">Internalized Stigma</td><td>1. I feel ashamed of being a refugee/immigrant.</td></tr>
<tr><td>2. Being a refugee/immigrant makes me feel that I am a bad person.</td></tr>
<tr><td>3. I feel I’m not as good as others because I am a refugee/immigrant.</td></tr>
<tr><td>4. I think less of myself because I am a refugee/immigrant.</td></tr>
<tr><td>5. I feel like it is my fault that I became a refugee/immigrant.</td></tr>
<tr><td>6. I deserve bad things for being a refugee/immigrant.</td></tr>
<tr><td>7. I feel less worthy because I am a refugee/immigrant.</td></tr>
<tr><td>8. I feel inferior to people who are not refugees/immigrants.</td></tr>
<tr><td rowspan="6">Perceived Stigma</td><td>1. Most people seem uncomfortable with refugees/immigrants.</td></tr>
<tr><td>2. Most people view refugees/immigrants as immoral.</td></tr>
<tr><td>3. Most people believe that refugees/immigrants should be isolated from society.</td></tr>
<tr><td>4. Most people don’t want to be friends with a refugee/immigrant.</td></tr>
<tr><td>5. Most people don’t want their children around refugees/immigrants.</td></tr>
<tr><td>6. Most people believe that refugees/immigrants are a threat to society (e.g., taking away jobs, taking advantage of resources, changing culture).</td></tr>
</table>

		7. Most people believe refugees/immigrants are dangerous/criminal
		8. Most people think that refugees/immigrants are inferior.
		9. Most people think that refugees/immigrants cannot be trusted.
		10. Most people think that refugees/immigrants should be blamed for their conditions.
		11. Most people feel that being a refugee/immigrant is a sign of personal failure.
		12. Most people believe that refugees/immigrants are lazy.
	Experienced Stigma	1. People stayed away from me because I am a refugee/immigrant
		2. I was denied access to certain areas because I am a refugee/immigrant
		3. People made fun of me due to being a refugee/immigrant
		4. I was blamed for being a refugee/immigrant
		5. I was denied opportunities in the workplace/school (e.g., acceptance, extracurricular activities, promotion, raise, opportunities to work with customers) because I am a refugee/immigrant
		6. I was treated badly by doctors and/or nurses in health care settings because I am a refugee/immigrant
		7. I was made to feel unwelcomed by Turkish people because I am a refugee/immigrant
		8. People acted as if I am not smart because I am a refugee/immigrant

		9. People acted as if they are afraid of me because I am a refugee/immigrant
		10. People acted as if I am dishonest because I am a refugee/immigrant
		11. I was insulted because I am a refugee/immigrant
		12. I was stared or pointed at (looked at intently) in public because I am a refugee/immigrant
	Anticipated Stigma	1. People will stay away from me because I am a refugee/immigrant
		2. People will make fun of me due to being a refugee/immigrant
		3. People will think it is my fault that I became a refugee/immigrant
		4. I will be denied opportunities in the workplace/school (e.g., acceptance, extracurricular activities, promotion, raise, opportunities to work with customers) because I am a refugee/immigrant
		5. I will be treated badly by doctors and/or nurses in health care settings because I am a refugee/immigrant
		6. I will be made to feel unwelcomed by Turkish people because I am a refugee/immigrant
		7. People will act as if I am not smart because I am a refugee/immigrant
		8. People will act as if they are afraid of me because I am a refugee/immigrant
		9. People will act as if I am dishonest because I am a refugee/immigrant
		10. I will be insulted because I am a refugee/immigrant

		11. I will be stared or pointed at (looked at intently) in public because I am a refugee/immigrant

Note 1. We have incorporated both terms "refugee" and "immigrant" within our items to encompass and address the experiences of both displaced populations.

Note 2. In the items, the terms "Refugee," "Immigrant," or "Asylum Seekers" can be employed interchangeably based on the specific status of the population under scrutiny.

Instructions for RSS use:

For Internalized Stigma Questions:

Think about the **last six months.** Please indicate to what extent you agree or disagree with the following statements.

For Perceived Community Stigma Questions:

Think about the **last six months**. Please indicate to what extent you agree or disagree with the following statements in terms of how _______ people perceive refugees/immigrants. (fill in the blank with the host country nationality)

For Experienced Stigma Questions:

How often did any of the following things happen to you in the **last six months** because you are a refugee/immigrant? These questions may be similar to other questions. Please remember that this section is about lived experiences. Please make sure you answer each question even if it seems similar to a previous question.

For Anticipated Stigma Questions:

For the following, please tell us how likely it is that _______ people will treat you in the following ways in the future because you are a refugee/immigrant. (fill in the blank with the host country nationality). These questions may be similar to other questions. Please remember that this section is about possible future experiences. Please make sure you answer each question even if it seems similar to a previous question.
