# Supplementary material for: Refugee stigma and its toll on mental health: development and validation of the refugee stigma scale (RSS)
Source: BMJ Glob Health. 2025 Nov 13;10(11):e017276. doi: 10.1136/bmjgh-2024-017276 (PMC12625852; doi:10.1136/bmjgh-2024-017276)
Supplement: online supplemental table 1 [file bmjgh-10-11-s002.docx]

**Table 1. (Supplementary)**

*Bivariate Correlations Among Stigma Subscales, Clinical Measures, Post-Migration Living Difficulties, and Contact Experiences with Host-country Citizens*

|  | M | SD | 1 | 2 | 3 | 4 | 5 | 6 | 7 | 8 | 9 |
| --- | --- | --- | --- | --- | --- | --- | --- | --- | --- | --- | --- |
| 1. IS | 2.14/2.78 | .81/1.01 | 1 |  |  |  |  |  |  |  |  |
| 2. PS | 2.76/3.34 | .92/.92 | .39^**^/.52^**^ | 1 |  |  |  |  |  |  |  |
| 3. ES | 2.18/2.70 | .85/.99 | .29^**^/.48^**^ | .42^**^/.67^**^ | 1 |  |  |  |  |  |  |
| 4. AS | 2.94/3.39 | .95/.96 | .39^**^/.55^**^ | .53^**^/.70^**^ | .70^**^/.77^**^ | 1 |  |  |  |  |  |
| 5. Depression | 2.23/2.61 | .76/.78 | .37^**^/.48^**^ | .27^**^/.53^**^ | .46^**^/.54^**^ | .37^**^/.55^**^ | 1 |  |  |  |  |
| 6. Anxiety | 1.97/2.20 | .74/.79 | .32^**^/.43^**^ | .25^**^/.45^**^ | .42^**^/.49^**^ | .33^**^/.51^**^ | .75^**^/.76^**^ | 1 |  |  |  |
| 7. SS | 2.42/2.60 | .93/1.07 | .25^**^/.30^**^ | .20^**^/.42^**^ | .36^**^/.39^**^ | .28^**^/.45^**^ | .70^**^/.74^**^ | .68^**^/.70^**^ | 1 |  |  |
| 8. PMLD | 2.43/3.10 | .79/.92 | .25^**^/.40^**^ | .30^**^/.50^**^ | .59^**^/.55^**^ | .47^**^/.55^**^ | .56^**^/.61^**^ | .46^**^/.53^**^ | .50^**^/.52^**^ | 1 |  |
| 9. CE | 3.23/2.95 | .91/.86 | -.21^**^/-.32^**^ | -.33^**^/-.41^**^ | -.47^**^/-.41^**^ | -.43^**^/-.46^**^ | -.29^**^/-.36^**^ | -.26^**^/-/35^**^ | -.21^**^/-.29^**^ | -.32^**^/-.31^**^ | 1 |
| 10. PTSD | 2.34/2.88 | .93/1.15 | .27**/.41** | .28**/.52** | .44**/ .54** | .35**/.54** | .74**/.81** | .62**/.71** | .57**/.70** | .53**/.59** | -.29**/-.32* |

Note. ^**^ = Correlation is significant at the.01 level (2-tailed)., ^*^ = Correlation is significant at the.05 level (2-tailed). IS = Internalized Stigma, PS = Perceived Community Stigma, ES = Experienced Stigma, AS = Anticipated Stigma, SS = Somatic Symptoms, PMLD = Post-Migration Living Difficulties, and CE = Contact Experiences; PTSD = Post-Traumatic Stress Disorder, the values before slash (/) belong to Syrians, *and the values after slash (/) belongs to Afghans*
